# Treatment with Micafungin in a Preterm Neonate with an Invasive *Candida parapsilosis* Infection after a Severe Terlipressin-Induced Skin Necrosis

**DOI:** 10.3390/pathogens10070890

**Published:** 2021-07-13

**Authors:** Domenico Umberto De Rose, Fiammetta Piersigilli, Bianca Maria Goffredo, Olivier Danhaive, Andrea Dotta, Cinzia Auriti

**Affiliations:** 1 Neonatal Intensive Care Unit, Medical and Surgical Department of Fetus, Newborn and Infant—“Bambino Gesù” Children’s Hospital IRCCS, 00165 Rome, Italy; domenico.derose@opbg.net (D.U.D.R.); andrea.dotta@opbg.net (A.D.); 2 Division of Neonatology, Cliniques Saint-Luc, Catholic University of Louvain, 1200 Brussels, Belgium; fiammetta.piersigilli@uclouvain.be (F.P.); olivier.danhaive@uclouvain.be (O.D.); 3 Biochemistry Laboratory, Department of Specialist Pediatrics, “Bambino Gesù” Children’s Hospital IRCCS, 00165 Rome, Italy; biancamaria.goffredo@opbg.net; 4 Division of Neonatology, San Francisco Benioff Children’s Hospital, University of California, San Francisco, CA 94158, USA

**Keywords:** *Candida parapsilosis*, terlipressin, preterm, preterm neonate, skin necrosis, skin grafts

## Abstract

*Candida parapsilosis* infections are increasingly reported in preterm neonates, but the optimal treatment remains uncertain. We report the clinical history of an extremely preterm neonate, who developed a devastating skin necrosis due to terlipressin administration, with subsequent superinfection by *Candida parapsilosis*. The infant underwent multiple curettages and skin grafts to resolve skin lesions and was treated with systemic micafungin administration at a high dose (8 mg/kg/day), with resolution of the fungal infection.

## 1. Introduction

Invasive Candida infections (ICIs) are a major cause of morbidity and mortality among very preterm infants [1]; *Candida albicans* is the most frequently occurring species [2], although *Candida parapsilosis* infections are increasingly reported in preterm neonates [3]. Despite the high minimum inhibitory concentration (MIC) necessary for *Candida parapsilosis* eradication, echinocandins have a prominent role when an ICI occurs in very preterm infants. These patients often carry a central vascular catheter in which *Candida parapsilosis* forms a biofilm, due to its adherence properties that favor attachment to skin and catheters [4]. Echinocandins allow infection resolution despite the presence of the biofilm, and also have very little effect on the immature renal function of these babies. Micafungin is the only echinocandin approved for neonates and young infants. Therapy with 8 mg/kg/day achieved a high response in a phase two study on 35 neonates with confirmed or suspected ICIs and medical and surgical underlying diseases [5]. Herein, we report the successful treatment of an invasive *Candida parapsilosis* infection in a seriously ill, very preterm infant with a wide devasting skin necrosis, achieved thanks to systemic micafungin administration, combined with multiple curettages and skin grafts.

## 2. Case Description 

**Figure 1:**
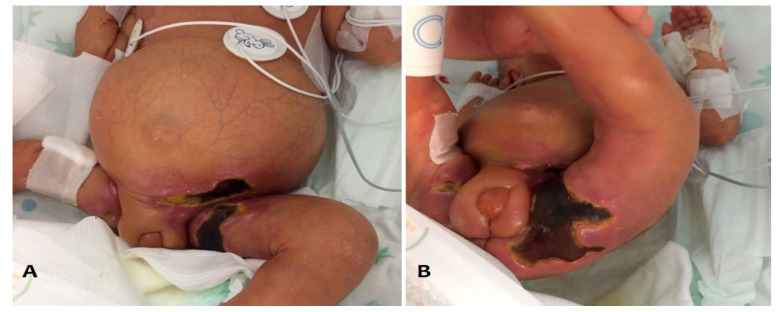
Indurative edema with violet stained skin of both thighs and perineal region (**A**) and severe necrotic skin lesions (**B**).

An extremely preterm neonate (gestational age, 24 weeks; body weight, 500 g) was born by vaginal delivery in a 2nd level hospital. She was intubated at birth and required surfactant administration and mechanical ventilation. Patent ductus arteriosus was closed with the administration of ibuprofen. Moreover the baby suffered from cholestasis, hypothyroidism treated with L-thyroxine (2 μg/kg/day), thrombosis of the inferior vena cava, requiring low-molecular-weight heparin, Clexane^®^,( marketed in Italy by Sanofi S.p.A.—Viale L. Bodio, 37/B—20158 Milano Italy) therapy and retinopathy of prematurity treated with photocoagulation. Antifungal prophylaxis with Fluconazole was started at birth, for a period of six weeks, then stopped on day of life 45. On day of life 63 (postmenstrual age: 33 weeks, weight: 1600 g), the neonate was still on mechanical ventilation due to severe bronchopulmonary dysplasia and pulmonary hypertension. An *Escherichia coli* sepsis occurred, and its condition worsened despite antibiotic treatment with imipenem, netilmicin and vancomycin. She developed persistent hypotension refractory to volume expanders, dopamine, epinephrine, vasopressin and norepinephrine. Therefore, therapy with terlipressin (triglycyl-lysine vasopressin, Glypressin^®^,( Ferring Pharmaceuticals Ltd.,Drayton Hall, Church Road. West Drayton, UB7 7PS, UK) dose: 20 μg/kg/h, was attempted and was administered through a well-positioned epicutaneo-cava catheter, placed through the right upper arm. After twenty days of continuous infusion, the baby showed indurative perineal edema, with a purple discoloration of the perineum and thigh teguments, quickly evolving into severe necrotic skin lesions (Figure 1). The neonate was, therefore, transferred to our neonatal intensive care unit (NICU) and the antifungal prophylaxis was already suspended.

Cutaneous swab and urine culture both yielded Candida parapsilosis. The fungal load in urine was 1,000,000 CFU/mL. Susceptibility tests were performed according to the Clinical and Laboratory Standards Institute (CLSI) guidelines [6,7]. Blood cultures resulted negative, but the child had been on antimicrobial therapy for several days. The isolated Candida strain was susceptible to micafungin (MIC, 0.5 µg/mL), anidulafungin (MIC, 2 µg/mL), fluconazole (MIC, 0.5 µg/mL), itraconazole (MIC, 0.06 µg/mL), voriconazole (MIC, <0.008 µg/mL) and amphotericin B (MIC, 0.5 µg/mL). Among the effective antifungals, we chose micafungin because of the feasibility of plasma concentration monitoring via heel stick capillary samples [8]. After given and obtained informed consent from the parents, both in relation to the high dosage of micafungin to be administered and monitored and to the participation in clinical trials and publications, micafungin was administered intravenously at a dose of 8 mg/kg/day; it was diluted in 0.9% saline solution at a concentration of 2 mg/mL and infused intravenously through epicutaneo-cava catheter in one hour. Plasma micafungin concentrations were measured by high performance liquid chromatography (HPLC) on the third day of the starting of therapy. The lower limit of detection (LLOD) of micafungin was 0.5 mg/L, and the lower limit of quantification (LLOQ) was 1 mg/L. (Table 1**)**.

With an 8 mg/kg/day micafungin dose, we were able to obtain adequate micafungin plasma concentrations pre-administration (on the third day of therapy plasma concentration was 3.73 mg/L), achieving a plasma Cmax/MIC ratio > 10. Although the first typical choice would have been catheter removal, we opted not to do so, because of the infant’s critical condition.

**Figure 2:**
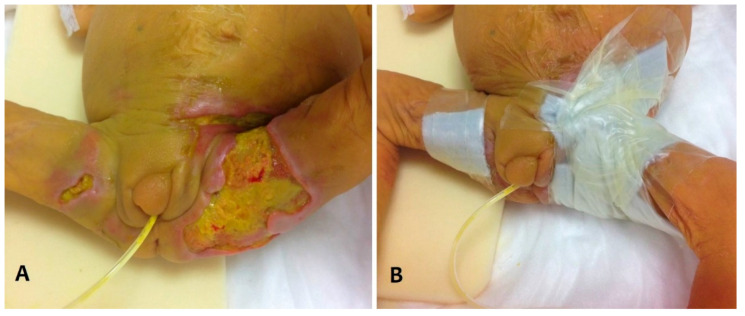
After surgical debridement of necrotic tissue (**A**), hydrocolloid dressing was applied twice weekly to stimulate skin healing (**B**).

ICI resolved after 15 days of treatment. Concurrently, after surgical debridement of necrotic tissue, hydrocolloid dressing was applied twice weekly to stimulate skin healing (Figure 2).

**Figure 3:**
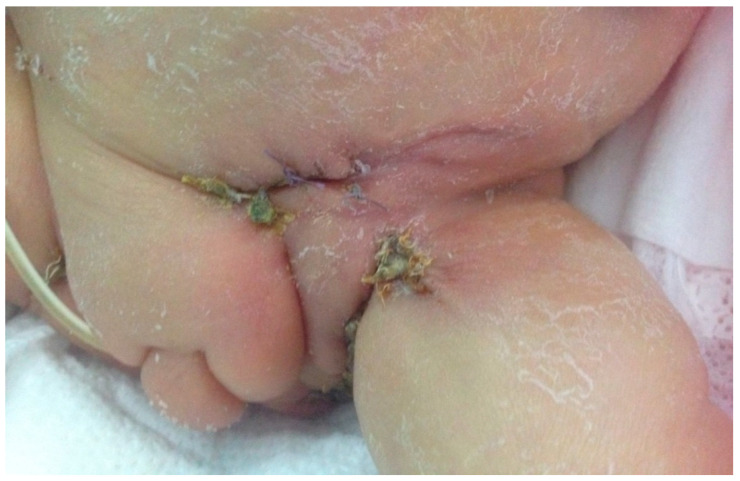
Gradual resolution of skin wounds after skin grafts.

Subsequently, cutaneous lesions were treated with two skin grafts (Figure 3). In order to allow the rapprochement with her parents, the child was transferred back to the hospital of origin, where she died after 2 months due to respiratory failure related to her severe bronchopulmonary dysplasia.

## 3. Discussion

We report an invasive *Candida parapsilosis* infection in an infant, who was born very preterm, with a wide devasting skin necrosis. The fungal infection was successfully treated with high-dose systemic micafungin. The baby required multiple curettages and skin grafts to treat the necrotic skin lesions, provoked by terlipressin infusion. Among non-albicans species, *Candida parapsilosis* is more frequent in neonates than in older children, given the wide use of intravascular devices in NICUs. Adherence properties of *Candida* parapsilosis favor adherence to skin and indwelling catheters, followed by biofilm formation [9]. Invasive *Candida spp*. infections in preterm neonates require a strong and early medical approach, given the high related mortality [10]. The most commonly used primary antifungal therapies for neonatal invasive candidiasis are fluconazole (32%), caspofungin (24%), liposomal amphotericin B (16%) and micafungin (8%), according to a large multicenter network [11].

Since, in our Unit Fluconazole is used as a prophylaxis, it is rarely and only in specific clinical situations administered as therapy to treat systemic fungal infections. Usually, the antifungal prophylaxis with fluconazole prevents the colonization of small patients hospitalized in NICU by the different species of *Candida*. To date, no resistance to this drug is reported and Fluconazole is the primary means of preventing fungal colonization in preterm infants. Probably in our patient, the fungal colonization occurred after the interruption of prophylaxis, in relation to prolonged antibiotic therapies, the patient’s long hospital stay, the site and extent of skin lesions.

Micafungin is the most studied echinocandin in neonates and the only one approved by both the European Medicine Agency (EMA) and United States Food and Drug Administration (US-FDA) for younger children, although the susceptibility of the *Candida parapsilosis* complex to echinocandins is not clear yet. In vitro activity of echinocandins seems to be adequate [12], but reduced echinocandin susceptibility has been reported in some strains [13].

Studies published so far still lack clear recommendations on recommendable micafungin dosages in preterm infants. It seems that these fragile patients require higher micafungin doses than older children and adults in order to reach therapeutic effects, due to increased plasma clearance [14]. Several studies demonstrated that high-dose micafungin is effective and safe in very preterm neonates also [15,16,17].

Given the severe clinical conditions of our patient, the epicutaneo-cava catheter was difficult to replace safely. The systemic therapy allowed complete infection control. Despite the baby’s previous cholestasis, therapy with micafungin did not cause side effects. We observed a transient increase in aspartate aminotransaminase and alanine aminotransaminase, which rapidly returned within normal range after discontinuation of therapy. We monitored plasma concentration of micafungin on 0.3 mL heel puncture blood samples, as previously described, in order to avoid excessive blood draws in a patient of low weight and in such severe clinical conditions [8].

Terlipressin is a synthetic long-acting analogue of vasopressin with a vasoconstrictor action. Its effects include significant increase in mean arterial pressure and systemic vascular resistance as well as significant decrease in heart rate, cardiac output, hepatic venous portal pressure gradient, and portal venous blood flow [18]. Although the actual efficacy of terlipressin in the neonatal period is still discussed [18], it can be used for the treatment of severe hypotension refractory to volume expansion, catecholamines, and corticosteroids. Skin necrosis has been described in adults as a rare side effect of terlipressin, presumably secondary to its long-acting vasoconstrictive effect [19,20,21]. To our knowledge, very few data are available to date regarding such severe complication in the neonatal period [22].

## 4. Conclusions

Micafungin, at a dose of 8 mg/kg/day, was effective and well tolerated in this extremely preterm, seriously ill neonate with an invasive *Candida parapsilosis* infection.

Although limited data exist about the use of terlipressin in neonates, skin necrosis seems to be an established severe side effect [22]. Given limited experience, its use should always be well weighed and limited only to life-threatening hypotensive shock.

## Figures and Tables

**Table 1:** Plasma micafungin concentrations (mg/L) evaluated using HPLC. Pre-administration value corresponds to the minimum plasma concentration (C min) of the time/concentration curve. The value one hour after infusion corresponds to the maximum plasma concentration (C max) of the time/concentration curve.

Dose (Over 24 h)	Site	30 min before Infusion	1 h after Infusion	2 h after Infusion	8 h after Infusion
8 mg/kg	Plasma	3.73	11.79	9.70	7.9

## Data Availability

Clinical data are available in the electronic archive of the Bambino Gesù Hospital and upon request to the author. Researchers may request access to anonymized participant-level data, trial-level data, and protocols from Astellas-sponsored clinical trials at www.clinicalstudydatarequest.com (accessed on 8 July 2021). For the Astellas criteria on data sharing, see https://clinicalstudydatarequest.com/Study-Sponsors/Study-Sponsors-Astellas.aspx (accessed on 8 July 2021).
